# Variability of the Left Atrial Appendage in Human Hearts

**DOI:** 10.1371/journal.pone.0141901

**Published:** 2015-11-06

**Authors:** Rafał Kamiński, Adam Kosiński, Mariola Brala, Grzegorz Piwko, Ewa Lewicka, Alicja Dąbrowska-Kugacka, Grzegorz Raczak, Dariusz Kozłowski, Marek Grzybiak

**Affiliations:** 1 Department of Clinical Anatomy, Medical University of Gdańsk, Gdańsk, Poland; 2 Department of Cardiology and Electrotherapy, Hospital of Medical University of Gdańsk, Gdańsk, Poland; University Hospital Medical Centre, GERMANY

## Abstract

Atrial fibrillation increases the risk of thrombus formation. It is commonly responsible for cerebral stroke whereas less frequently for pulmonary embolism. The aim of the study was to describe the morphology of the left atrial appendage in the human heart with respect to sex, age and weight. Macroscopic examination was carried out on 100 left appendages taken from the hearts of the patients aged 18–77, both sexes. All hearts preserved in 4% water solution of formaldehyde carried neither marks of coronary artery disease nor congenital abnormalities. Three axes of appendage orientation were performed. After the appendage had been cut off, morphological examination was performed in long and perpendicular axes. Measurements of the appendages were taken from anatomical specimens and their silicone casts. We classified the left atrial appendage into 4 morphological groups according to the number of lobes. Most left atrial appendages in female population were composed of 2 lobes. In the male group typically 2 or 3-lobed appendages were observed. The mean left atrial appendage orifice ranged from 12.0 to 16.0 mm and the most significant difference in the orifices between males and females was observed in LAA type 2 (about 3.3 mm). A smaller orifice and narrower, tubular shape of the LAA lobes could explain a higher risk of thrombus formation during nonvalvular atrial fibrillation in women. Knowledge of anatomical variability of the LAA helps diagnose some undefined echoes in the appendage during transesophageal echocardiographic examination.

## Introduction

Atrial fibrillation (AF) is the most common arrhythmia in the world, characterized by the mechanical dysfunction of both atria and their appendages. It affects about 1–2% of the general population [1,2] with estimated prevalence of 3 million people in the United States [3]. AF significantly increases the risk of thrombus formation in both appendages and is frequently responsible for cerebral stroke while rarely for pulmonary embolism [4]. The risk of embolic stroke incident is five-fold higher in people with AF [5]. The presence of thrombus in both appendages can be examined by transesophageal echocardiography (TEE) [6]. In literature, we can find some cases demonstrating difficulties with evaluation of the contents in the left atrial appendage (LAA) by TEE imaging [7]. LAA anatomy with different shapes and lobes has been regarded as a primary source of blood stasis and thrombus formation [8] and can correlate with the risk of stroke in patients with AF [9]. Currently, oral anticoagulation therapy is the most effective prophylactic approach in patients with AF and increased risk of thromboembolic events [10]. Unfortunately, only 50–60% of patients taking Coumadin (warfarin) fall into the therapeutic range, thus effective, long-term anticoagulation is very difficult [11]. New oral anticoagulant drugs like: dabigatran, rivaroxaban, apixaban–are the alternative to warfarin, but they are still associated with potential bleeding complications and there is no antagonist agent in case of bleeding events [12].

## Aim

The aim of our study was evaluation of the left atrial appendage morphology in human hearts. Morphometric measurements of both heart appendages and their silicone casts were analysed by sex, heart’s weight and age in two groups: below 60 years of age (Y), and above 60 years of age (O).

## Material and Methods

**Fig 1:**
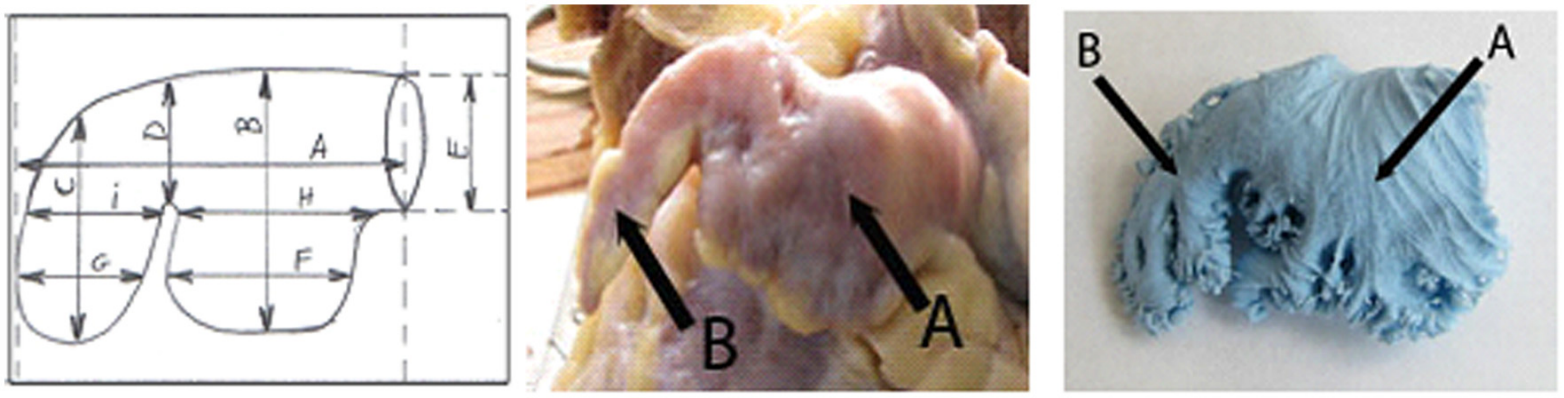
LAA type 1: A diagram, an anatomical sample and a silicone cast. A-proximal lobe, B-distal lobe.

**Fig 2:**
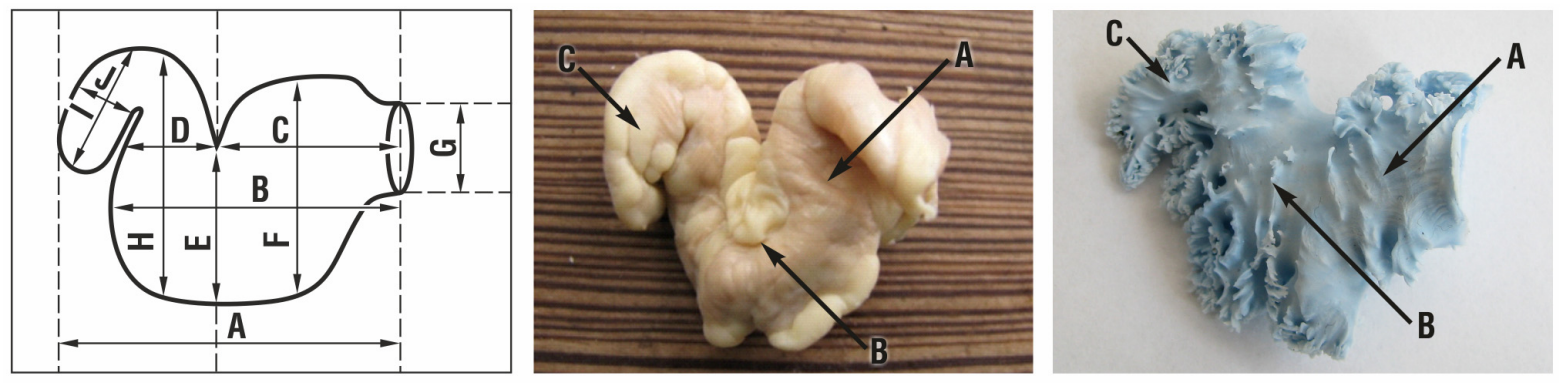
LAA type 2: A diagram, an anatomical sample and a silicone cast. A-proximal lobe, B-middle lobe, C-distal lobe.

**Fig 3:**
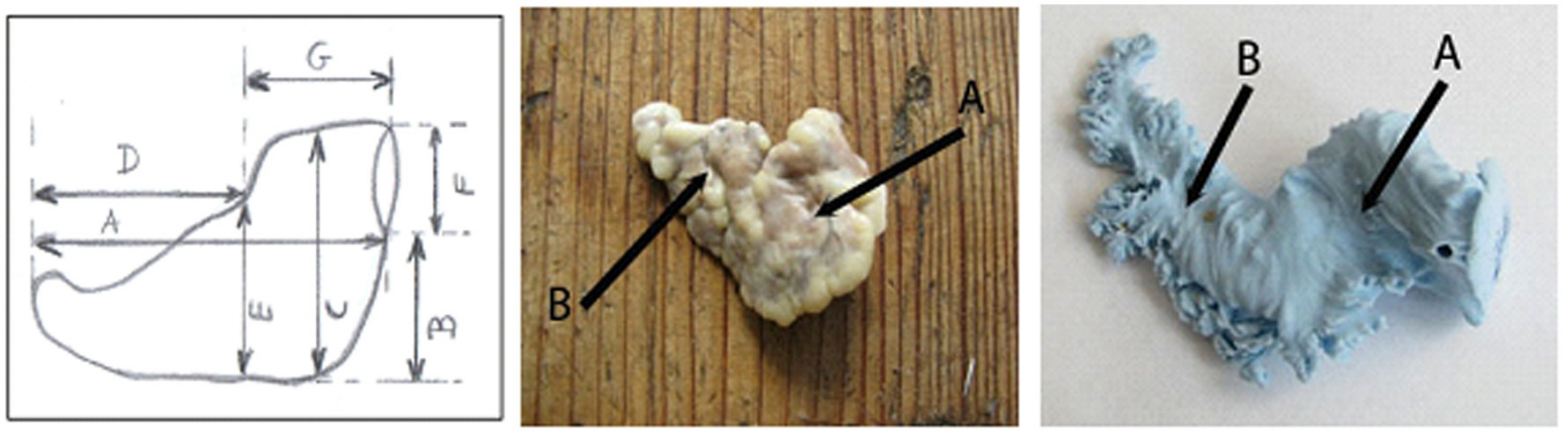
LAA type 3: A diagram, an anatomical sample and a silicone cast. A-proximal lobe, B-distal lobe.

**Fig 4:**
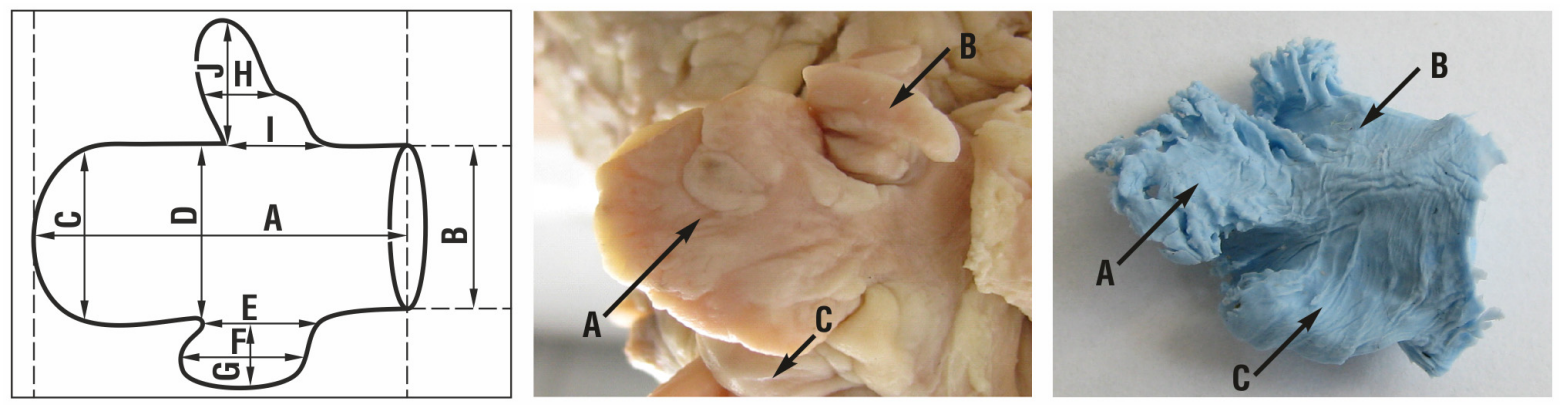
LAA type 4: A diagram, an anatomical sample and a silicone cast. A-central lobe, B-superior lobe, C- inferior lobe.

Our research project was approved by Independent Research Bioethics Committee of Medical University of Gdańsk (IRBC) and achieved permit number: NKEBN/40-119/2010. Investigation has been conducted according to the principles expressed in the Declaration of Helsinki. The decision of IRBC repeals the need for the consent of donors and their next of kin for the implementation of the research presented in the article. IRBC specifically waived the need for written informed consent from the donors/next of kin. Macroscopic examination was carried out on 100 left appendages (Y-62, O-38) taken from the hearts of both sexes aged 18–77. In all cases, clinical data invariably indicated a negative history of cardiovascular disease and death due to noncardiac causes. The hearts evaluated via gross examination showed no evidence of pathologies. Subsequently, the hearts were preserved in 4% water solution of formaldehyde. The hearts weighted from 210–490 grams. Classical anatomical studies were applied. After removing the pericardium we evaluated the shape of the left atrial appendage (LAA). Because of the LAA shape variability, we proposed to distinguish 4 types of the appendage (Table 1). Type 1 was composed of the proximal and lower-distal lobe (Fig 1), type 2 had three lobes–proximal, middle and distal (Fig 2), type 3 –proximal and upper-distal lobe (Fig 3) and type 4 –central lobe and superior and inferior lobe/lobes (Fig 4). For the purpose of macroscopic examination we assigned 3 axes of LAA. The long axis was a dimension from the base to the apex of the appendage, the short axis was a distance between upper and lower edge of the appendage and the perpendicular axis was between both walls of the appendage. After the appendage had been cut off, morphological examination was performed and external measurements were taken in the long and short axes. To evaluate the internal measurements of the LAA we prepared their silicone casts by means of silicone mass (MM 922, catalyser B-5). All data were analysed by the R statistical program.

**Table 1:** General classification of the LAA morphology.

LAA t.1	The dominant lobe extended from the left atrium towards the anterio-lateral direction. The distal part characterized itself as a narrow tube with its apex facing down
LAA t.2	The dominant lobe (proximal) extended from the lateral wall of the left atrium and descended towards anterio-lateral direction. The middle lobe folded up towards the base of the heart and bent down again to form a distal lobe
LAA t.3	The proximal lobe extended from the anterio-lateral part of the left atrium wall and ran slightly down, next it continued into the distal lobe with its apex facing up and bent towards the beginning of the aorta and the pulmonary trunk
LAA t.4	The main trunk of appendage (central lobe) started from anterio-lateral part of the left atrium wall and run in horizontal plane towards the anterior surface of the heart. On the upper and lower edge of the main lobe most commonly two small lobes were found:superior and inferior

## Results

### Left atrial appendage type 1

Atrial appendage occurred in 56% examined hearts and was composed of two parts. The dominant lobe extended from the left atrium towards the anterio-lateral direction. The distal part characterized itself as a narrow tube with its apex facing down (Fig 1). The ostium of the proximal lobe to the left atrium was smaller in females than in males and measured: 18.8 and 19.7 mm, respectively. In both groups, the size of the proximal lobe was similar. The distal lobe was longer in females by about 3 mm and narrower by about 1 mm. Moreover, in people over 60 years of age the distal lobe was longer by about 2.8 mm compared to younger group. The ostium size in the older group was bigger compared to the younger one and measured 17.7 and 22.9 mm, respectively (Table 2).

**Table 2:** The comparison of the mean dimensions (mm) measured in LAA type 1 analysed by the sex and the age.

Dimensions	A	B	C	D	E	F	G	H	I
LAA 1	32.5±5.3	23.1±4.1	18.1±5.8	12.0±4.2	19.4±4.2	21.0±4.5	9.8±2.5	19.7±4.1	10.5±2.8
Females	33.2±6.3	23.1±3.9	20.1±5.8	13.2±6.0	18.8±3.3	20.6±2.0	9.1±3.1	19.6±2.0	10.0±3.4
Males	32.1±4.6	23.1±4.2	17.0±5.6	11.3±2.7	19.7±4.6	21.2±5.5	10.2±2.1	19.7±4.9	10.8±2.5
Group < 60	29.0±4.1	22.7±4.4	16.7±3.6	10.2±2.5	17.7±4.4	20.3±2.0	8.8±2.7	19.7±2.2	9.7±2.5
Group > 60	34.3±4.6	24.5±2.8	19.5±7.6	11.6±2.8	22.9±2.6	23.2±7.8	8.7±1.3	22.6±5.5	9.9±1.2

### Left atrial appendage type 2

Atrial appendage type 2 was present in 23% examined hearts and was composed of 3 lobes. The dominant lobe (proximal) extended from the lateral wall of the left atrium and descended towards anterio-lateral direction. The middle lobe folded up towards the base of the heart and bent down again to form a distal lobe. In the female group the ostium was smaller than in the male one by about 3.6 mm and measured 12.0 and 15.6 mm, respectively. In the female group all lobes of the LAA t.2 were smaller. Moreover both the connections between proximal and middle lobes and between middle and distal lobes were narrower by over 7 and 3.6 mm, respectively. In the older group the distal lobe was longer and wider by 4.7 and 3.4 mm, respectively. The proximal lobe did not differ between the younger and the older group (Table 3).

**Table 3:** The comparison of the mean dimensions (mm) measured in LAA type 2 analysed by the sex and the age.

Dimensions	A	B	C	D	E	F	G	H	I	J
LAA t.2	33.9±2.2	26.5±2.1	14.6±2.3	10.0±1.6	16.6±3.7	20.5±3.0	15.3±3.2	20.6±4.2	15.7±4.0	8.3±2.1
Females	30.0±1.4	25.0±1.4	12.0±1.4	9.0±1.4	10.0±1.4	16.0±1.4	12.0±1.4	17.0±1.4	9.0±1.4	5.0±1.4
Males	34.2±1.9	26.7±2.1	14.9±2.2	10.1±1.6	17.2±3.2	21.0±2.7	15.6±3.2	21.0±4.3	16.4±3.5	8.6±1.9
Group < 60	32.2±2.7	26.1±1.4	13.5±1.5	11.0±1.9	13.8±4.7	20.4±3.6	16.0±4.5	21.0±5.0	13.0±4.5	7.0±1.5
Group > 60	35.2±1.2	25.0±1.0	13.6±0.9	9.4±1.3	18.7±1.3	20.4±1.1	16.3±1.9	18.4±3.4	17.7±3.6	10.4±1.1

### Left atrial appendage type 3

Left atrial appendage type 3 occurred in 13% of the examined hearts and was composed of 2 lobes. The proximal lobe extended from the anterio-lateral part of the left atrium wall and ran slightly down, next it continued into the distal lobe with its apex facing up and bent towards the beginning of the aorta and the pulmonary trunk. Generally, the female appendage was bigger compared to the male one. The connection between the appendage and the left atrium (orifice) in females was smaller by about 1.3 mm compared to males. The proximal lobe was significantly bigger in females by about 2.2 mm in the short axis and 6.4 mm in the long axis, contrary to the distal part which was shorter in that group. Because of few LAA t.3 in the examined material of the hearts, we were unable to make a comparison between the hearts above and over 60 years of age (Table 4).

**Table 4:** The comparison of the mean dimensions (mm) measured in LAA type 3 analysed by the sex and the age.

Dimensions	A	B	C	D	E	F	G
LAA t.3	31.0±3.8	18.0±3.2	29.0±3.0	19.0±1.8	22.0±3.5	14.0±1.3	12.0±2.3
Females	33.6±3.2	19.4±3.0	30.4±3.1	18.5±2.1	24.5±2.3	13.5±1.2	15.1±1.4
Males	28.2±1.5	15.3±1.9	28.2±2.5	19.5±1.0	19.2±2.3	14.8±1.2	8.7±1.2

### Left atrial appendage type 4

Left atrial appendage type 4 was the rarest one and was observed only in 8% of the hearts. It was composed mainly of 3 parts. The main trunk of the appendage (central lobe) started from the anterio-lateral part of the left atrium wall and descended (ran) in horizontal plane towards the anterior surface of the heart. On the upper and lower edge of the main lobe most commonly two small lobes were found: superior and inferior. In the female population the central lobe was bigger contrary to the male group: both in the long and short axes but the ostium of the appendage was 1.5 mm smaller in that group. The superior lobe was 3 mm bigger in the short axis in the female group compared to male one, with the similar dimension in the long axis. The inferior lobe was bigger in females in all dimensions (Table 5).

**Table 5:** The comparison of the mean dimensions (mm) measured in LAA type 4 analysed by the sex and the age.

Dimension	A	B	C	D	E	F	G	H	I	J
LAA t.4	30.0±1.8	17.9±1.1	11.3±2.1	12.0±2.6	20.4±1.5	18.3±4.2	9.0±2.8	7.9±0.7	16.3±3.6	12.3±2.1
Females	31.3±0.6	17.0±1.0	11.7±0.6	12.7±0.6	21.7±0.6	22.7±0.6	11.7±0.6	7.7±0.6	20.0±0.0	14.0±1.0
Males	29.0±1.8	18.5±0.6	11.0±2.9	11.5±2.3	19.5±1.3	15.0±0.8	7.0±1.8	8.0±0.8	13.5±1.3	11.0±1.8

The most frequent type of LAA in males was type 1, which occurred in 54% of the hearts. LAA type 2 was observed in 31%, LAA type 3 –in 9% and the rarest LAA type 4 was noticed only in 6% of the hearts. In the female group the most common appendage was type 1, it occurred in 61%, next one was LAA type 3–24%, LAA type 4–9% and the most infrequently occurring one—LAA type 2 observed only in 6% (Table 6).

**Table 6:** The comparison of the incidence of LAA types.

	LAA t.1	LAA t.2	LAA t.3	LAA t.4
Females	61%	6%	24%	9%
Males	54%	31%	9%	6%

On the basis of our study we noticed that in the female group, the connection between the left atrium and the appendage was smaller in all types of LAA. The smallest difference between the sizes of the orifices in both groups was observed in LAA type 1, whereas the biggest one was in LAA type 2 (Table 7).

**Table 7:** The size of the ostium (mm) in all types of LAA in females and males.

	LLA t.1	LAA t.2	LAA t.3	LAA t.4
Females	15.6	9.0	11.4	13.1
Males	16.1	12.3	13.0	15.0
Difference	0.5	3.3	1.6	1.9

## Discussion

In medical journals there are few publications describing gross morphology of the left atrial appendage. Studies presenting LAA taken from a CT image reconstruction are most common. Generally, LAA is described as a tubular, narrow, hooked and finger-like structure [12,13] or a pouch–like extension of the body of the atrium [14]. On the basis of 220 LAA casts examinations Ernst et al. observed variability of LAA [15]. They described the course and ramifications of the principal axis of the LAA and the orifice diameters. The course of the principal axis was straight (7%), slightly bent and slightly spiral (23%), slightly bent and extremely spiral (5%), extremely bent and slightly spiral (24%) and extremely bent and extremely spiral (42%). The mean orifice diameters for LAA with >5 branches and >40 twigs were 15.0 and 21.0 mm, respectively. The first, “lobe classification” of LAA morphology was published in 1997 in *Circulation* by Veinot et al.[16]. They determined the orifice diameter, width, length and the number of lobes, unfortunately they did not describe the relation of the lobes in all types of LAA. They distinguished 4 types of LAA. The dominant type was the appendage composed of two lobes which occurred in 54% of the hearts, three-lobed LAA existed in 23%, one-lobed appendage was observed in 20% and the rarest one, composed of 4 lobes was found only in 3% of the examined hearts. Moreover they found the orifice size to be age- and sex-related. In men and women over 20 years old, the mean orifice diameters of 11.6 and 10.7 mm were found, respectively. On the basis of our research we also found LAA orifice diameters to be sex-related. In women we observed a smaller orifice size in all types of LAA. Comparing female and male groups we noticed that the biggest difference in the orifice size was in LAA type 2 and accounted for 9.0 and 12.3 mm, respectively. This feature can be useful for determination of a proper size of the occluder device placed in the LAA as a prevention of stroke in patients with nonvalvular AF (nvAF),[17,18,19]. In other types of LAA there were no so significant differences in the orifice size: LAA t.1–0.5 mm; LAA t.3–1.6 mm; LAA t.4 -1.9 mm. Su *et al*.[20] determined LAA orifice to be mostly oval in shape, not round. The longer dimension was 17.4±4 mm and the shorter one– 10.9 ± 4.2 mm. In our work we studied only the longer dimension of the LAA orifice and it measured from 12 to 16 mm, depending on the LAA type. In the studies which were carried out in living patients (Transesophageal Echocardiography or Computed Tomography), the orifice size was bigger. Nucifora *et al*. [21] presented LAA in 3-dimential TEE and they showed that the longer dimension of LAA orifice measured 22.3 ± 4.5 mm, and shorter one– 16.5 ± 4.3 mm. Moreover they pointed out that in patients with paroxysmal or persistent AF the orifice of the LAA was bigger and it changed its shape from oval to more roundish. The differences in the LAA orifice sizes observed in post-mortem and alive examination were the caused by formaldehyde, affecting the heart tissue and time of preservation. According to our study LAA type 1 was very often observed in male and female hearts. The main difference in the appendage between both groups was the distal lobe which was narrower and longer in women. That tubular shape of the distal lobe, which gets longer, especially in women over 60 years old may determine a different course of the coagulation process during nvAF. The analysis of the LAA type 2 occurring more commonly in the male population showed that it was definitely larger than in women. Moreover, the orifice and proximal-middle lobe connections were significantly smaller in females, which can be conducive to thrombus formation, too. LAA type 3 was more commonly observed in females than in males. Also, the appendage was longer in women with the smaller orifice to the left atrium. Such morphological features of the left atrial appendage may increase the risk of ischaemic stroke in women. Available clinical data suggest a higher thromboembolism (TE) rate in women. CHA_2_DS_2_-VASc score predicting TE events reflects this finding by providing risk stratification scheme which takes into account the sex of patients with atrial fibrillation,[22,23,24]. New advanced visualization techniques of the heart allow us to perform evaluation of the LAA. Using a computed tomography and reconstruction of the images, Wang et.al formed a new classification of LAA (Table 8),[25]. In our study, we generally focused on the main, invariable lobes which compose LAA not on the twigs or small ramifications. Comparing our results to the Veinot’s classification we observed similar incidence of LAA types. The most frequent type of LAA was the two-lobed appendage (type 1 and type 3): 69%, the three-lobed LAA was found in 23% examined hearts. WindSock and ChickenWing LAA in Wang’s classification existed in 66% and fulfilled the criteria of our LAA type 1and type 3 which were observed in 69%. Our research showed that the most common types of LAA in the female group were type 1 and type 3 (85% of all female hearts), but in themale group the dominant types of LAA were type 1 and type 2 (85% of all male hearts). This showed that in women we can mostly find 2-lobed LAA but in men mostly 2- and 3-lobed LAA. Moreover, the Ucerler’s et al. study showed that the most common type of LAA was the 2-lobed appendage [26]. Summing up, in our population we can mostly find 2-lobed left atrial appendage. This knowledge could be useful in some unclear cases during TEE examination when thrombus or “normal”anatomy of the LAA have to be differentiated [7].

**Table 8:** Wang’s classification of left atrial appendage.

*Chicken Wing*	with an obvious bend in the proximal or middle part of the dominant lobe,or folding back of the LAA anatomy on itself at some distance from the perceived LAA ostium. this type of the LAA may have the secondary lobes or twigs
*Wind Sock*	with one dominant lobe of sufficient length as the primary structure. variations of this LAA type arise with the location and number of secondary or even tertiary lobes arising from the dominant lobe
*Cauliflower*	with limited overall length with more complex internal characteristics. Variations of this LAA type have a more irregular shape of the LAA ostium and a variable number of lobes with lack of a dominant lobe
*Cactus*	with dominant central lobe with secondary lobes extending from the central lobe in both superior and inferior directions

## Conclusion

The study results confirmed considerable morphological differences in the LAA. A significant variability was found in the appendage shape and the dimensions of the structures observed–with the tendency to reach lower values in the hearts in women. In the majority of cases, both in males and females, the appendages had 2 lobes, while 3-lobed appendages were less common and mainly found in men. This anatomical variability seems to be clinically significant and may affect the course of the coagulation process inside the appendage, and thus may be the cause of the diagnostic errors when TEE is performed.
